# Rac3 Expression and its Clinicopathological Significance in Patients With Bladder Cancer

**DOI:** 10.3389/pore.2021.598460

**Published:** 2021-03-30

**Authors:** Mei Chen, Zhenyu Nie, Hui Cao, Yuanhui Gao, Xiaohong Wen, Chong Zhang, Shufang Zhang

**Affiliations:** ^1^ Central Laboratory, Affiliated Haikou Hospital of Xiangya Medical College, Central South University, Haikou, China; ^2^ Urology, Affiliated Haikou Hospital of Xiangya Medical College, Central South University, Haikou, China

**Keywords:** Rac3, bladder cancer, TCGA, GSEA, prognose, clinicopathological significance

## Abstract

**Background:** Ras-related C3 botulinum toxin substrate 3 (Rac3) is overexpressed in malignancies and promotes tumor progression. However, the correlations between Rac3 expression and the clinicopathological characteristics and prognoses of patients with bladder cancer (BC) remain unclear.

**Methods:** Data from The Cancer Genome Atlas (TCGA) were used to analyze Rac3 expression in BC and normal bladder tissues and validated using the Oncomine database, quantitative real-time PCR (qRT-PCR) and western blot. The Kaplan-Meier method was used to analyze the relationship between Rac3 expression and the prognosis of patients with BC. Cox univariate and multivariate analyses of BC patients overall survival (OS) were performed. Signaling pathways that potentially mediate Rac3 activity in BC were then analyzed by gene set enrichment analysis (GSEA).

**Results:** The Rac3 expression in BC tissues was significantly higher than that in normal bladder tissues. Rac3 expression was significantly correlated with grade and stage. Overexpression of Rac3 was associated with a poor prognosis. GSEA showed that the cell cycle, DNA replication, p53 signaling pathway and mismatch repair were differentially enriched in the high Rac3 expression phenotype. The qRT-PCR and western blot results confirmed that the Rac3 expression in BC tissues was higher than that in normal bladder tissues.

**Conclusion:** Rac3 is highly expressed in BC, which is related to the advanced clinicopathological variables and adverse prognosis of patients with BC. These results provide a new therapeutic target for BC.

## Introduction

Bladder cancer (BC) is one of the most common malignant tumors in the urogenital system [1] and is pathologically divided into muscle-invasive bladder cancer (MIBC) and nonmuscle invasive bladder cancer (NMIBC) [2]. NMIBC accounts for approximately 75% of all BC patients cases, and 10–30% of patients have a poor prognosis and can further develop MIBC [3, 4]. MIBC has high rates of malignancy, recurrence and mortality and high metastatic potential. BC treatment is expensive [5]. Although many treatments are available, the patient prognosis is poor, and the curative effect is not satisfactory [6]. The morbidity and mortality rates of BC in males are higher than those in females, and most of these BC cases originate from the urinary epithelium [4]. The high recurrence rate and drug resistance of BC after surgery are challenging aspects of clinical research and new biomarkers are urgently needed to improve personalized treatment for patients with BC [7]. Therefore, it is important to find and study genes related to the occurrence and development of BC to thus guide the treatment and prognostic prediction of BC. Currently, no effective biomarkers are available to predict adverse prognosis in BC.

Ras-related C3 botulinum toxin substrate 3 (Rac3) is a member of the Rho GTPase family [8]. Rho and Rac GTPases can promote cancer progression [9], and have a wide range of cellular roles, including regulating cell migration and adhesion, mitosis, and kinase activity [10]. The Rac3 gene is located on chromosome 17q23–25. Rac3 is active in combination with GTP and unactive in combination with GDP. When Rac3 is activated, it is able to stimulate efficiently the c-Jun amino-terminal kinase signaling pathway [11]. It has been proven that Rac3 is highly expressed in many tumors, such as prostate cancer [12] and brain tumors [13]. However, Rac3 expression in BC has rarely been reported.

In this research, we first evaluated the expression of Rac3 in patients with BC and studied its relationships with the clinicopathological variables and overall survival (OS) of patients with BC. Second, we analyzed the biological processes and signal transduction pathways that may mediate Rac3 activity in BC, which provides new ideas for the role of Rac3 in BC.

## Materials and Methods

### Data Sources

The BC cohort in The Cancer Genome Atlas (TCGA) database contained 408 BC patients. The RNA-seq data of patients with BC standardized by the fragments per kilobase of transcript per million fragments mapped (FPKM) and the corresponding clinical and prognostic information were downloaded from the official TCGA website (https://cancergenome.nih.gov/). The inclusion criteria for patients in our study were as follows: 1) Rac3 expression values and complete clinicopathological information including the age, gender, grade, stage, and N stage; and 2) overall survival (OS). Patients with a survival time of 0 months were excluded from our study. Finally, we selected 357 patients with BC for further analysis (Table 1). The Lee Bladder [14] and Sanchez-Carbayo Bladder 2 [15] datasets were obtained from the Oncomine database (https://www.oncomine.org) to verify the Rac3 mRNA expression in BC tissues and normal controls. GSE32894 was downloaded from the Gene Expression Omnibus (GEO) database (https://www.ncbi.nlm.nih.gov/geo/) to analyze the association between Rac3 expression and the prognosis of patients with BC. We collected the clinicopathologic information from the three datasets (Supplementary Tables S1–S4).

**TABLE 1:** Clinical characteristics of patients with BC in the TCGA database.

Characteristics		Total	%	Rac3 expression values
Age at diagnosis (y)		62 (34–89)		6.40 (0.42–123.16)
Gender	Male	263	73.67	6.41 (0.42–123.16)
	Female	94	26.33	6.26 (0.66–105.29)
Grade	High	339	94.96	6.64 (0.42–123.16)
	Low	18	5.04	4.55 (1.19–12.82)
Stage	I	1	0.28	3.37
	II	100	28.01	5.51 (0.98–76.22)
	III	130	36.42	7.06 (0.42–123.16)
	IV	126	35.29	7.17 (1.62–68.32)
N stage	N0	234	65.55	6.12 (0.42–123.16)
	N1	43	12.04	7.37 (1.62–68.32)
	N2	73	20.45	6.29 (1.62–46.46)
	N3	7	1.96	15.53 (6.83–31.36)

BC, bladder cancer; TCGA, the Cancer genome atlas; N, lymph node metastasis; GO, gene ontology; KEGG, kyoto encyclopedia of genes and genomes analyses of genes coexpressed with Rac3.

The genes coexpressed with Rac3 were retrieved and extracted using the multi experiment matrix (MEM; https://biit.cs.ut.ee/mem/index.cgi) and cBioPortal databases (http://www.cbioportal.org/). The coexpressed genes in the two databases were then overlapped in a Venn diagram (http://bioinfogp.cnb.csic.es/tools/venny/). These coexpressed genes were subjected to GO and KEGG enrichment analyses using the database for annotation, visualization and integrated discovery (DAVID; https://david.ncifcrf.gov/). The critical value for significant gene enrichment functions was set at *p* < 0.05.

### Gene Set Enrichment Analysis

GSEA is a computational method used to determine whether a group of genes defined a priori shows statistical significance between two biological states [16]. GSEA was performed using GSEA3.0 (http://www.broad.mit.edu/gsea/), and patients with BC were divided into a high-expression group and a low-expression group according to the median Rac3 expression level. The c2.cp.kegg.v6.2. symbols.gmt dataset was obtained from the Molecular Signatures database (MSigDB) on the GSEA website. Enrichment analysis was carried out by default weighted enrichment statistics, and the analyses were randomly repeated 1,000 times. Nominal *p* < 0.05 and a false discovery rate (FDR) < 0.25 were used as the cutoff criteria.

### Patients and Human Tissue Specimens

BC tissues and adjacent normal bladder tissues were collected from 8 patients undergoing BC surgery at the Affiliated Haikou Hospital of Xiangya Medical College, Central South University, in 2019. We compiled the clinical and pathological characteristics of the 8 BC patients (Supplementary Table S5). Consent from all patients and approval from the organizational ethics committee were obtained before collection.

### Quantitative Real-Time Polymerase Chain Reaction

Total RNA and cDNA were obtained with TRIzol Reagent (Vazyme, Nanjing, China) and TransScript One-Step gDNA Removal (Vazyme) according to the manufacturers’ instructions. cDNA was amplified by qRT-PCR on a LightCycler 480II system using SYBR qPCR Master Mix (Vazyme). The oligonucleotide sequences of the primers were as follows: Rac3 (forward: 5′-CCT​CCT​TCG​AGA​ATG​TTC​GT-3′ and reverse: 5′-AGG​TAT​TTC​ACA​GAG​CCA​ATC​T-3′) and GAPDH (forward: 5′-CAG​GAG​GCA​TTG​CTG​ATG​AT-3′ and reverse: 5′-GAA​GGC​TGG​GGC​TCA​TTT-3′). Rac3 mRNA expression was calculated using the 2^−ΔΔct^ method, and each experiment was repeated three times.

### Western Blot Analysis

Fresh frozen tissue samples were homogenized in lysis buffer (Solarbio, Beijing, China) and quantified with an Easy Protein Quantitative Kit (TransGen Biotech, Beijing, China). Fifty micrograms of total protein lysate was loaded in each lane. Proteins were separated by 10% sodium dodecyl sulfate polyacrylamide gel electrophoresis (SDS-PAGE) and transferred to PVDF membranes (Biosharp, Guangzhou, China). The PVDF membranes were incubated with anti-Rac3 (1:1,000; ab124943, Abcam, Cambridge, United Kingdom) and anti-GAPDH (1:2,000; Bioss, Beijing, China) antibodies overnight at 4°C. Then, the membranes were incubated with the secondary antibodies horseradish peroxidase (HRP)-conjugated goat anti-rabbit IgG (1:2,000; ab205718, Abcam, United States) and goat anti-mouse IgG (1:2,000; ab205719, Abcam, United States) at room temperature for 1.5 h. The membranes were visualized using an EasySee® Western Blot Kit (TransGen Biotech). The experiment was performed three separate times, and two parallel samples were ran in each experiment.

### Statistical Analysis

All statistical analyses were performed using R software (version 3.5.1) and GraphPad Prism (version 7.0). The difference in Rac3 expression between BC tissues and normal bladder tissues was analyzed by the *t*-test. The Wilcoxon signed-rank test was used to analyze the relationships between Rac3 and the clinicopathological characteristics of patients with BC. The log-rank and Gehan-Breslow-Wilcoxon tests in the Kaplan-Meier method were used to analyze the OS of patients with BC. Cox univariate and multivariate analyses were performed to assess the OS of patients with BC. Signifcance was set at *p* < 0.05.

## Results

### Overexpression of Rac3 in BC Tissues by Dataset Analyses

**FIGURE 1:**
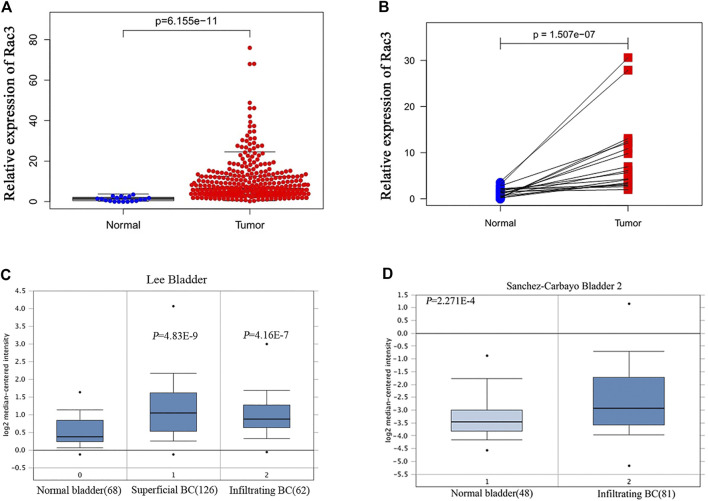
High Rac3 expression in BC as determined by dataset analyses. **(A)** Rac3 expression in unpaired BC and normal bladder tissues in the TCGA cohort. **(B)** Rac3 expression in paired BC tissues and normal bladder tissues in the TCGA cohort. Rac3 mRNA expression profile in the Oncomine database: **(C)** Lee Bladder; **(D)** Sanchez-Carbayo Bladder 2.

The results of RNA-seq data analysis of TCGA databases showed that the Rac3 expression in BC tissues was higher than that in normal bladder tissues (*p* = 6.155*e* − 11) (Figure 1A). Considering individual differences, further analysis of 18 paired tissues showed that the Rac3 expression in BC tissues was significantly higher than that in normal bladder tissues (*p* = 1.507*e* − 07) (Figure 1B). In the Oncomine database, the “Lee Bladder” dataset contains 126 superficial BC tissue samples, 62 invasive BC samples and 68 normal tissue samples, and the “'Sanchez-Carbayo Bladder 2” dataset contains 81 invasive BC samples and 48 normal tissue samples. Data from the Oncomine database confirmed that the Rac3 expression in BC tissues (269 cases) was higher than that in normal bladder tissues (116 cases, *p* < 0.001) (Figures 1C,D).

### Correlation Between Rac3 Expression and Clinicopathological Variables of BC Patients

**FIGURE 2:**
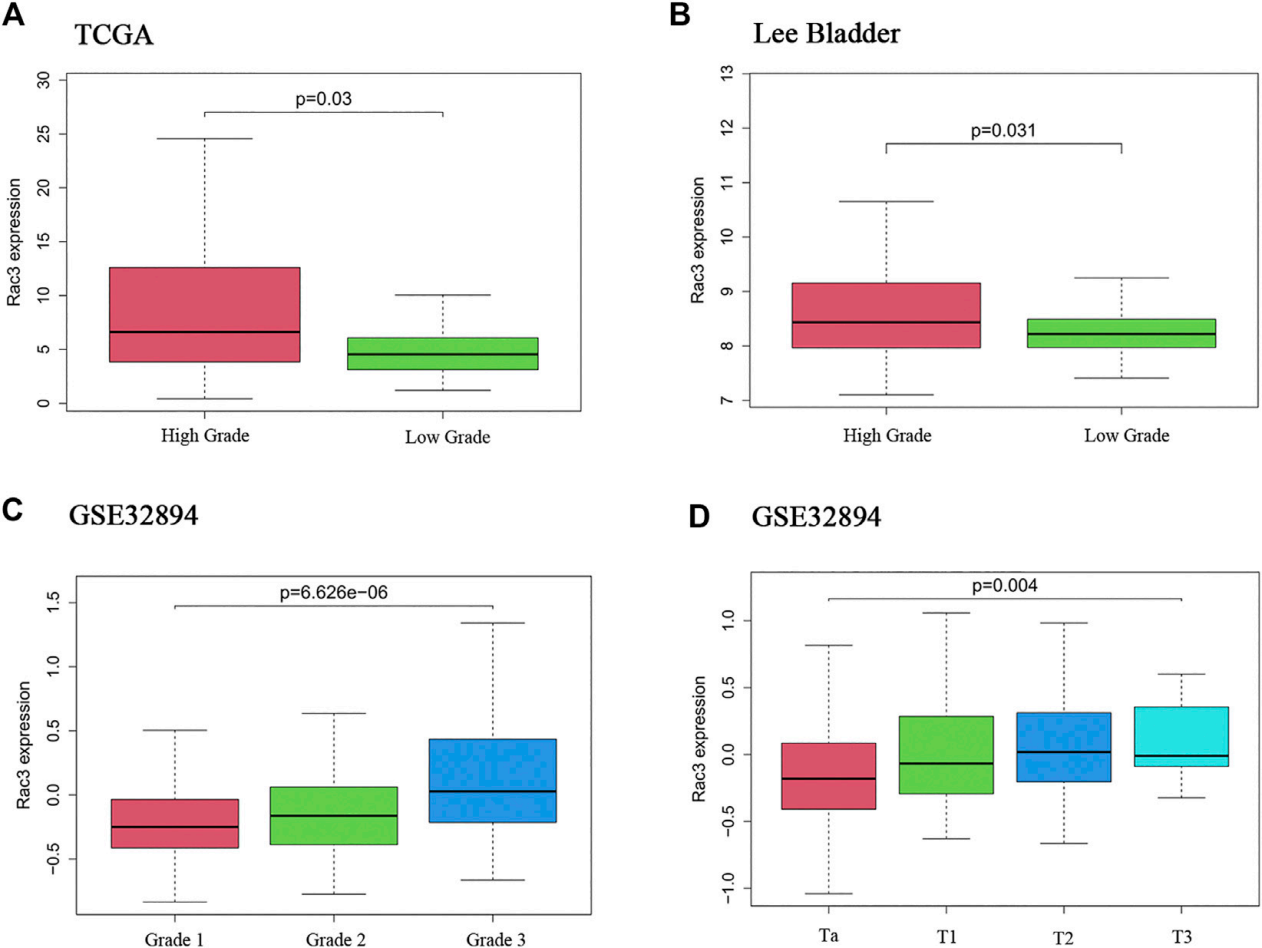
Correlations between Rac3 expression and clinicopathological variables. **(A)** Grade in the TCGA databases (WHO 2004). **(B)** Grade in the “Lee Bladder” dataset (WHO 2004). **(C)** Grade in the GSE32894 dataset (WHO 1999). **(D)** Stage in the GSE32894 dataset. Correlation between Rac3 expression and the prognosis of patients with BC.

To analyze the correlations between Rac3 expression and the clinicopathological variables of patients with BC, we collected clinical data from 357 patients with BC from the TCGA database. Rac3 expression was significantly associated with the tumor grade (*p* = 0.03) (Figure 2A), as its expression increased with the grade. In the 'Lee Bladder' dataset, we also found that the expression of Rac3 was significantly associated with the tumor grade (*p* = 0.031) (Figure 2B). In the GSE32894 dataset, we found that expression of Rac3 was significantly associated with the tumor grade (*p* = 6.626*e* − 06) (Figure 2C) and stage (*p* = 0.004) (Figure 2D).

**FIGURE 3:**
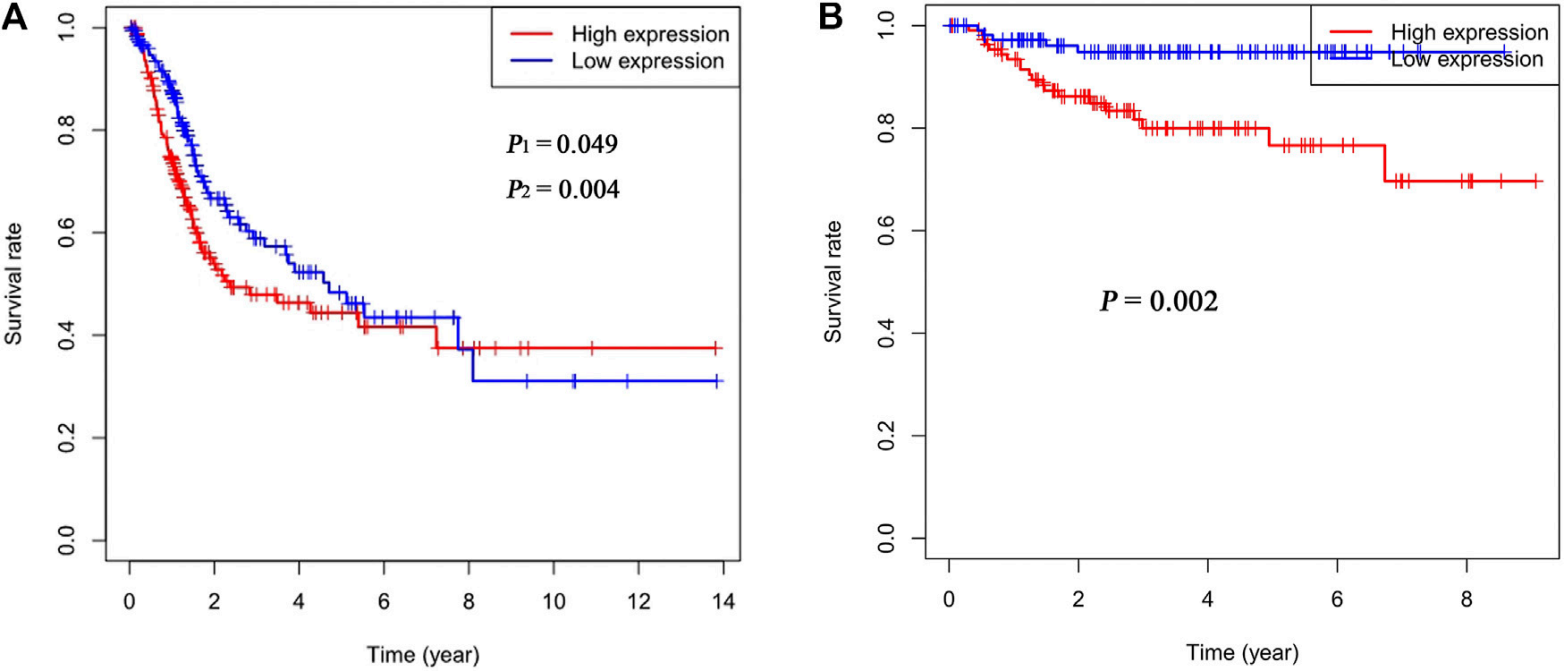
Correlation between Rac3 expression and the OS of patients with BC **(A)** TCGA database. **(B)** GSE32894 dataset. *p*_1_ was determined by the log-rank test, and *p*_2_ was determined by the Gehan-Breslow-Wilcoxon test.

The cutoff value for Rac3 expression was 6.40 in the TCGA database, and patients were divided into a high-expression group and a low-expression group based on this value. Kaplan-Meier survival analysis showed that patients with high Rac3 expression had a worse prognosis than those with low expression over the short term (range 1–95 months) (log-rank test, *p*_1_ = 0.049, Gehan-Breslow-Wilcoxon test, *p*_2_ = 0.004) (Figure 3A). Cox univariate analyses showed that Rac3 expression was associated with poor prognosis (hazard ratio [HR]: 1.280, 95% confidence interval [CI] = 1.091–1.502, *p* = 0.002), and other clinical variables associated with poor survival included age, tumor stage and N stage. Cox multivariate analyses showed that Rac3 was independently associated with OS (HR: 1.019, 95% CI = 1.007–1.031, *p* = 0.002), and other factors affecting OS were age and tumor stage (Table 2). In the GSE32894 dataset, Kaplan-Meier survival analysis also showed that patients with high Rac3 expression had a worse prognosis than those with low expression (*p* = 0.002) (Figure 3B).

**TABLE 2:** Cox univariate and multivariate analyses of the OS of patients with BC in the TCGA database.

Clinical variables	Univariate analysis			Multivariate analysis		
	HR	95% CI	*p* Value	HR	95% CI	*p* value
Rac3	1.280	1.091–1.502	0.002	1.019	1.007–1.031	0.002
Age	1.040	1.021–1.059	2.31*e* − 05	1.035	1.017–1.054	0.000
Gender	0.896	0.617–1.301	0.564	—	—	—
Stage	1.941	1.529–2.464	5.05*e* − 08	1.522	1.037–2.232	0.032
N stage	1.617	1.358–1.924	6.35*e* − 08	1.212	0.896–1.637	0.211

BC, bladder cancer; OS, overall survival; N, lymph node metastasis; Rac3, ras-related C3 botulinum toxin substrate 3; HR, hazard ratio; CI, confidence interval.

### Cluster Analyses of Genes Coexpressed With Rac3

**FIGURE 4:**
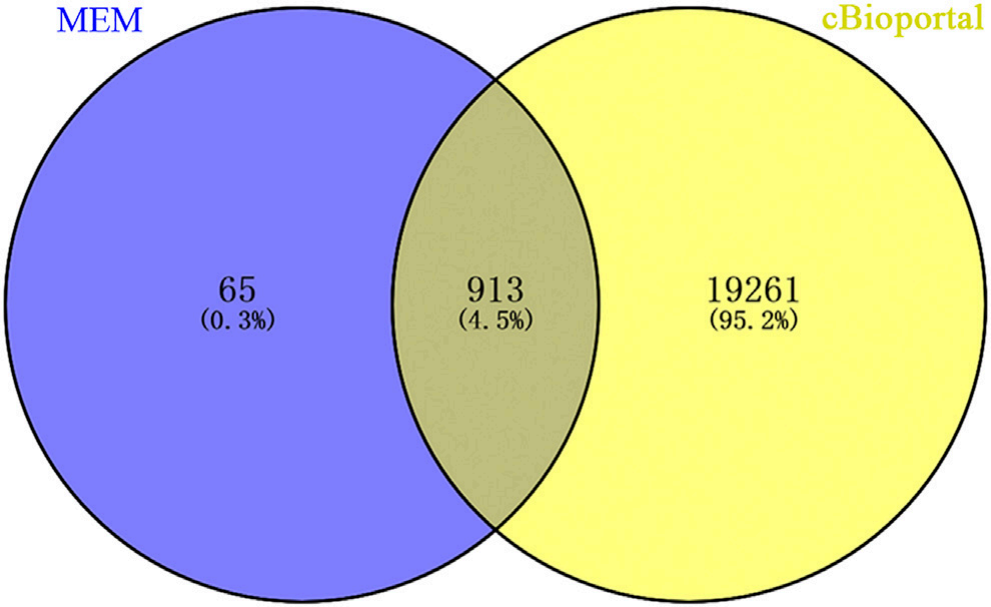
Venn diagram showing the overlap of coexpressed genes between MEM and cBioPortal. Abbreviation: MEM: multi experiment matrix.

A total of 978 genes coexpressed with Rac3 were extracted from the MEM with at least two independent gene probes. Additionally, 20,177 genes were coexpressed with Rac3 in cBioPortal. The coexpressed genes obtained from the above databases were intersected to obtain 913 genes for further analysis (Figure 4).

**FIGURE 5:**
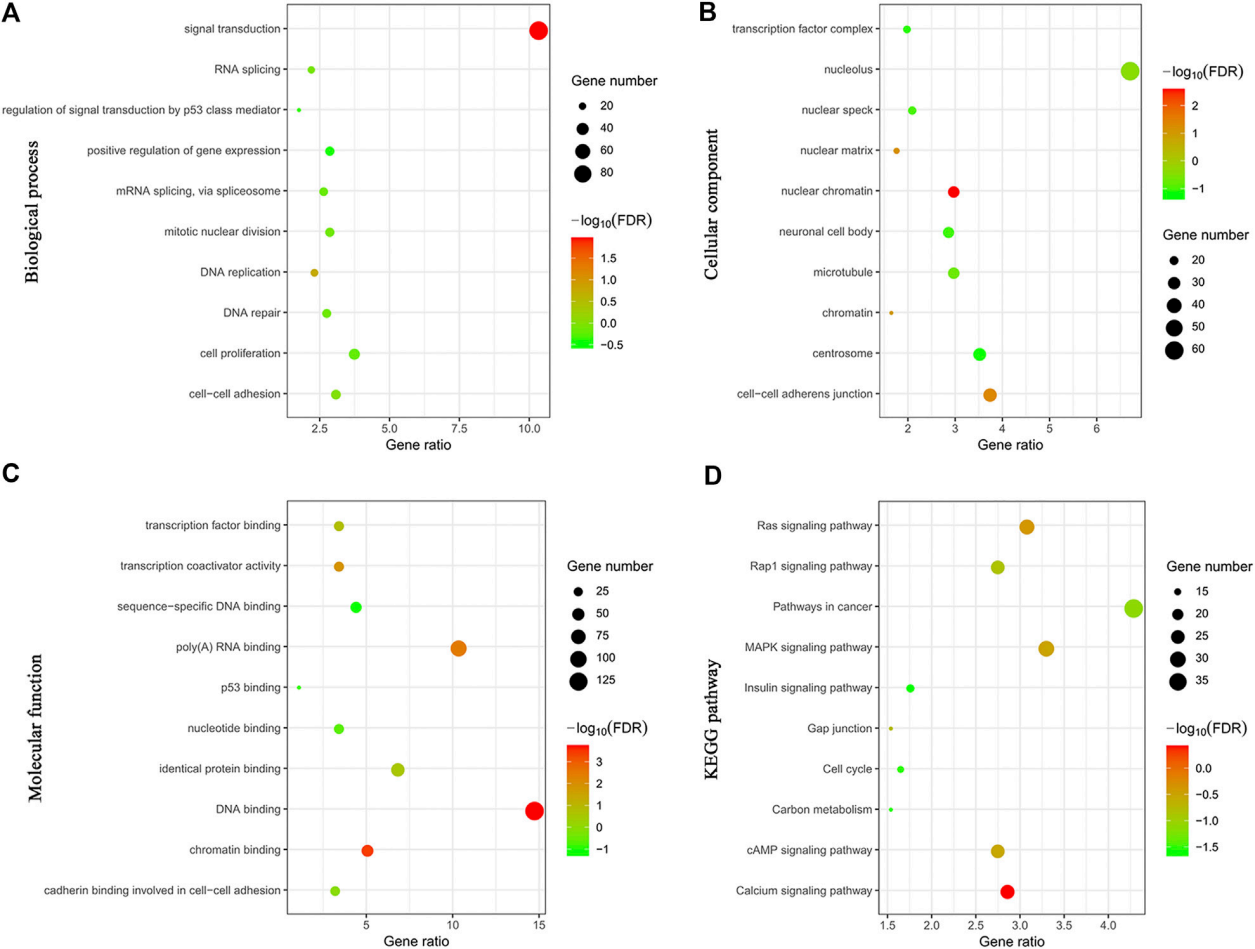
Cluster analyses of genes coexpressed with Rac3. **(A)** Biological process. **(B)** Cellular component. **(C)** Molecular function. **(D)** KEGG. FDR, false discovery rate.

To explore the potential mechanism of Rac3 in BC, genes coexpressed with Rac3 were used to predict the function of Rac3. The 913 coexpressed genes were analyzed by GO and KEGG with DAVID. The results indicated that these coexpressed genes were mainly enriched in signal transduction, regulation of signal transduction by p53 class mediator, positive regulation of gene expression, DNA replication, DNA repair and cell proliferation in the biological process category (Figure 5A), in the transcription factor complex and nucleolus in the cellular component category (Figure 5B), and in transcription, sequence-specific DNA binding, p53 binding and identical protein binding in the molecular function category (Figure 5C). KEGG analysis revealed that the genes coexpressed with Rac3 were mainly enriched in the cell cycle, pathways in cancer, Ras signaling and insulin signaling pathways (Figure 5D).

**FIGURE 6:**
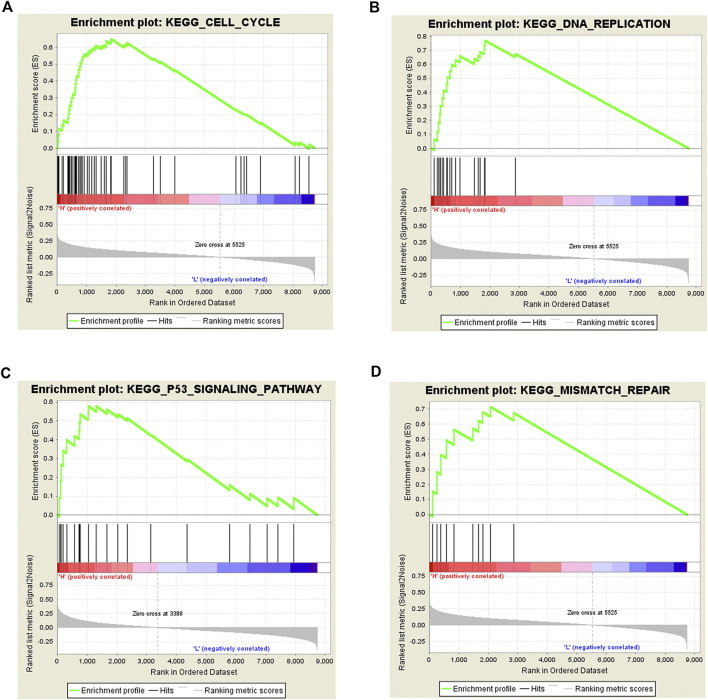
Gene set enrichment analysis of Rac3 in BC. **(A)** cell cycle, **(B)** DNA replication, **(C)** p53 signaling pathway, and **(D)** mismatch repair were differentially enriched with the phenotype of high Rac3 expression.

To explore the possible pathways by which Rac3 affects BC, we performed GSEA. The results showed that the cell cycle, DNA replication, p53 signaling pathway and mismatch repair were differentially enriched with the phenotype of high Rac3 expression (Table 3) (Figure 6), which was basically consistent with the pathway analysis of coexpressed genes. These results suggested that Rac3 may be associated with the cell cycle, DNA replication, p53 signaling pathway and mismatch repair. GSEA results provide good insight into the mechanisms of Rac3 in BC.

**TABLE 3:** The enriched pathways associated with Rac3 expression.

Gene set	ES	NES	NOM *p*-val	FDR *q*-val
Cell cycle	0.65	2.34	≤0.001	0.000
DNA replication	0.77	2.29	≤0.001	0.000
p53 signaling pathway	0.58	1.71	0.027	0.137
Mismatch repair	0.71	1.75	0.001	0.030

ES, enrichment score; NES, normalized enrichment score; NOM, nominal; FDR, false discovery rate.

### Validation of Rac3 Expression by qRT-PCR and Western Blot

**FIGURE 7:**
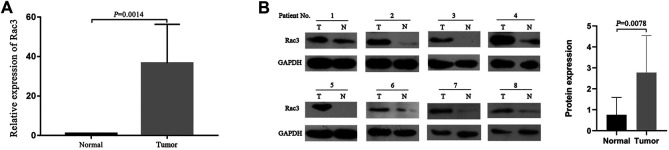
Rac3 expression in BC tissues. **(A)** The Rac3 mRNA expression was analyzed in paired tumor and normal bladder tissues by qRT-PCR. **(B)** The Rac3 protein levels were measured in cancer tissues and normal bladder tissues from 8 patients with BC by western blot. Mean and standard deviations (SD) are indicated in the figures. Abbreviations: T: tumor tissue, N: normal bladder tissues.

Finally, we analyzed the Rac3 mRNA and protein levels in 8 pairs of BC and normal bladder tissues by qRT-PCR and western blot. Compared with those in normal bladder tissues, both the Rac3 mRNA (*p* = 0.0014) (Figure 7A) and protein expression (*p* = 0.0078) (Figure 7B) were increased in BC tissues.

## Discussion

Members of the Rho GTPase family are related to tumorigenesis and are important regulators of cellular functions [17]. Many studies have reported the expression and function of Rac3 in cancers, such as esophageal [17], breast [18–21] and gastric cancer [22]. but its expression and function in BC are still unclear. Recently, some studies have used databases to analyze the prognostic relevance of Rac3 in BC [23–27], but strict inclusion criteria were in place for the patients with BC in this study, and the number of patients we analyzed was different. We first performed Kaplan-Meier survival analysis, followed by univariate and multivariate Cox analysis. Qiu et al. [23] and Liu et al. [24] screened only prognostic immune-related genes with univariate Cox analysis, while Na et al. [25] and Tang et al. [26] used prognostic immune-related genes for multivariate Cox analysis. However, we used Rac3 and clinicopathological variables for multivariate Cox analysis. Therefore, the results we obtained are different.

The TCGA project, launched by the United States in 2005, aims to apply genomic analysis techniques to study genomic changes in cancer to enable early diagnosis, treatment and prevention of cancer. Oncomine is a large database of cancer gene chips, representing 65 gene chip datasets, 4,700 chips, and expression data for 480 million genes. In this study, data from the above databases were used to determine that Rac3 is highly expressed in BC and that its expression is related to advanced clinicopathological variables and can predict poor prognosis. Rac3 expression was shown to be higher in lung adenocarcinoma tissues than in normal tissues and was an independent risk factor for N stage, which was associated with poor survival [28]. The activation of Rac3 is associated with an invasive and metastatic phenotype of breast cancer cells [19]. siRNA-mediated depletion of Rac3 strongly inhibits the invasive behaviors of glioblastoma and breast carcinoma cells [18]. However, the effect of Rac3 on the biological function of BC cells needs to be verified by experiments.

With the development of high-throughput sequencing and new computing methods, accumulating evidence shows that multiple genes interact with one another and influence the occurrence and development of tumors. Wang et al. [29] used KEGG to identify significant pathways for the differential expression of coding genes and their subsets. To investigate the function of Rac3, we carried out GO and KEGG analyses of genes coexpressed with Rac3. The biological processes enriched by genes coexpressed with Rac3 include signal transduction, regulation of signal transduction by p53 class mediator, DNA replication, DNA repair, mitotic nuclear division and cell proliferation. KEGG pathway analysis showed that genes coexpressed with Rac3 were significantly enriched in the cell cycle and pathways in cancer. GSEA can be used to elucidate biological pathways in which genes are involved [30]. Cantini et al. [31] used GSEA to identify signaling pathways modulated in cell lines upon the silencing of single microRNAs. Menzl et al. [32] used GSEA to identify mTOR signaling pathway that is perturbed in the absence of CDK8 and experimentally confirmed that CDK8 plays an important role in the mTOR signaling pathway. GSEA using TCGA data further showed that the cell cycle, DNA replication, p53 signaling pathway and mismatch repair were differentially enriched with the phenotype of high Rac3 expression. We found that genes coexpressing with Rac3 were associated with signal transduction, DNA replication, cell proliferation, the cell cycle and pathways in cancer, and we speculate that Rac3 may also be related to these pathways. Furthermore, we found that Rac3 was associated with the cell cycle, DNA replication and p53 signaling pathway as determined by GSEA. The cell cycle is reportedly involved in the development of cancer [33]. Liu et al. [34] found that knockdown of Rac3 led to G2/M phase cell cycle arrest and excess cell accumulation in the G1 and S phases. Wang et al. [35] found that silencing Rac3 could significantly inhibit cell growth, reduce colony formation, arrest the cell cycle and promote apoptosis in lung adenocarcinoma. The Rac effector p21-activated kinase (Pak) is associated with active Rac3, the Rac3-Pak pathway is critical for DNA synthesis, and hyperactive Rac3 controls the proliferation of breast cancer cells by a p21-activated kinase-dependent pathway [20]. Long noncoding RNAs (lncRNAs) regulate the p53 signaling pathway in BC [29, 36]. TMEM40 plays an important role in proliferation and apoptosis via the p53 signaling pathway in BC [37]. The study also has some limitations. We were unable to provide information regarding the cellular biological functions and mechanisms of Rac3 in BC, and the sample size was too small. Thus, more experiments are needed to validate these findings in the future.

## Conclusion

In this research, we found that Rac3 is highly expressed in BC and is a potential prognostic marker in patients with BC. Our study provides a good idea for the mechanism of Rac3 in BC. However, experiments are needed to explain the molecular mechanisms and functions underlying the promotion of BC development by Rac3.

## Data Availability

The datasets used and/or analyzed during the current study are available from TCGA, Oncomine and GEO databases.

## References

[1] Ferlay J Shin H-R Bray F Forman D Mathers C Parkin DM. Estimates of worldwide burden of cancer in 2008: GLOBOCAN 2008. Int J Cancer (2010). 127(12):2893–917. 10.1002/ijc.25516 21351269

[2] Samaratunga H Makarov DV Epstein JI. Comparison of WHO/ISUP and WHO classification of noninvasive papillary urothelial neoplasms for risk of progression. Urology (2002). 60(2):315–9. 10.1016/s0090-4295(02)01705-3 12137833

[3] Siegel RL Miller KD Jemal A. Cancer statistics, 2017. CA: A Cancer J Clinicians (2017). 67(1):7–30. 10.3322/caac.21387 28055103

[4] Kamat AM Hahn NM Efstathiou JA Lerner SP Malmström P-U Choi W Bladder cancer. The Lancet (2016). 388(10061):2796–810. 10.1016/s0140-6736(16)30512-8 27345655

[5] Johnson DC Greene PS Nielsen ME. Surgical advances in bladder cancer. Urol Clin North America (2015). 42(2):235–52. 10.1016/j.ucl.2015.01.005 25882565

[6] Chavan S Bray F Lortet-Tieulent J Goodman M Jemal A. International variations in bladder cancer incidence and mortality. Eur Urol (2014). 66(1):59–73. 10.1016/j.eururo.2013.10.001 24451595

[7] Bruchbacher A Soria F Hassler M Shariat SF D’Andrea D. Tissue biomarkers in nonmuscle-invasive bladder cancer. Curr Opin Urol (2018). 28(6):584–90. 10.1097/mou.0000000000000546 30188332

[8] Van Aelst L D'Souza-Schorey C. Rho GTPases and signaling networks. Genes Develop (1997). 11(18):2295–322. 10.1101/gad.11.18.2295 9308960

[9] Fritz G Just I Kaina B. Rho GTPases are over-expressed in human tumors. Int J Cancer (1999). 81(5):682–7. 10.1002/(sici)1097-0215(19990531)81:5<682::aid-ijc2>3.0.co;2-b 10328216

[10] Onesto C Shutes A Picard V Schweighoffer F Der CJ. Characterization of EHT 1864, a novel small molecule inhibitor of Rac family small GTPases. Methods Enzymol (2008). 439:111–29. 10.1016/s0076-6879(07)00409-0 18374160

[11] Haataja L Groffen J Heisterkamp N. Characterization of RAC3, a novel member of the Rho family. J Biol Chem (1997). 272(33):20384–8. 10.1074/jbc.272.33.20384 9252344

[12] Engers R Ziegler S Mueller M Walter A Willers R Gabbert HE. Prognostic relevance of increased Rac GTPase expression in prostate carcinomas. Endocr Relat Cancer (2007). 14(2):245–56. 10.1677/erc-06-0036 17639041

[13] Hwang S-L Chang J-H Cheng T-S Sy W-D Lieu A-S Lin C-L Expression of Rac3 in human brain tumors. J Clin Neurosci (2005). 12(5):571–4. 10.1016/j.jocn.2004.08.013 15993075

[14] Lee J-S Leem S-H Lee S-Y Kim S-C Park E-S Kim S-B Expression signature of E2F1 and its associated genes predict superficial to invasive progression of bladder tumors. Jco (2010). 28(16):2660–7. 10.1200/jco.2009.25.0977 20421545

[15] Sanchez-Carbayo M Socci ND Lozano J Saint F Cordon-Cardo C. Defining molecular profiles of poor outcome in patients with invasive bladder cancer using oligonucleotide microarrays. JCO (2006). 24(5):778–89. 10.1200/jco.2005.03.2375 16432078

[16] Subramanian A Tamayo P Mootha VK Mukherjee S Ebert BL Gillette MA Gene set enrichment analysis: a knowledge-based approach for interpreting genome-wide expression profiles. Proc Natl Acad Sci (2005). 102(43):15545–50. 10.1073/pnas.0506580102 16199517 PMC1239896

[17] Dong S Zhao J Wei J Bowser RK Khoo A Liu Z F-box protein complex FBXL19 regulates TGFβ1-induced E-cadherin down-regulation by mediating Rac3 ubiquitination and degradation. Mol Cancer (2014). 13:76. 10.1186/1476-4598-13-76 24684802 PMC3994216

[18] Chan AY Coniglio SJ Chuang Y-Y Michaelson D Knaus UG Philips MR Roles of the Rac1 and Rac3 GTPases in human tumor cell invasion. Oncogene (2005). 24(53):7821–9. 10.1038/sj.onc.1208909 16027728

[19] Baugher PJ Krishnamoorthy L Price JE Dharmawardhane SF. Rac1 and Rac3 isoform activation is involved in the invasive and metastatic phenotype of human breast cancer cells. Breast Cancer Res (2005). 7(6):R965–R974. 10.1186/bcr1329 16280046 PMC1410764

[20] Mira J-P Benard V Groffen J Sanders LC Knaus UG. Endogenous, hyperactive Rac3 controls proliferation of breast cancer cells by a p21-activated kinase-dependent pathway. Proc Natl Acad Sci (2000). 97(1):185–9. 10.1073/pnas.97.1.185 10618392 PMC26637

[21] Gest C Joimel U Huang L Pritchard LL Petit A Dulong C Rac3 induces a molecular pathway triggering breast cancer cell aggressiveness: differences in MDA-MB-231 and MCF-7 breast cancer cell lines. BMC Cancer (2013). 13:63. 10.1186/1471-2407-13-63 23388133 PMC3576359

[22] Pan Y Bi F Liu N Xue Y Yao X Zheng Y Expression of seven main Rho family members in gastric carcinoma. Biochem Biophysical Res Commun (2004). 315(3):686–91. 10.1016/j.bbrc.2004.01.108 14975755

[23] Qiu HD Hu XR He C Yu BB Li YQ Li JN. Identification and validation of an individualized prognostic signature of bladder cancer based on seven immune related genes. Front Genet (2020). 11:12. 10.3389/fgene.2020.00012 32117435 PMC7013035

[24] Liu L Hu J Wang Y Sun T Zhou X Li X Establishment of a novel risk score model by comprehensively analyzing the immunogen database of bladder cancer to indicate clinical significance and predict prognosis. Aging (2020). 12(12):11967–89. 10.18632/aging.103364 32570217 PMC7343485

[25] Na L Bai Y Sun Y Wang Z Wang W Yuan L Identification of 9-core immune-related genes in bladder urothelial carcinoma prognosis. Front Oncol (2020). 10:1142. 10.3389/fonc.2020.01142 32733809 PMC7360854

[26] Tang Y Hu Y Wang J Zeng Z. A novel risk score based on a combined signature of 10 immune system genes to predict bladder cancer prognosis. Int Immunopharmacology (2020). 87:106851. 10.1016/j.intimp.2020.106851 32763782

[27] Cheng CY Song DK Wu YD Liu BQ. RAC3 promotes proliferation, migration and invasion via PYCR1/JAK/STAT signaling in bladder cancer. Front Mol biosciences (2020). 7:218. 10.3389/fmolb.2020.00218 PMC7488983 33062641

[28] Zhang C Liu T Wang G Wang H Che X Gao X Rac3 regulates cell invasion, migration and EMT in lung adenocarcinoma through p38 MAPK pathway. J Cancer (2017). 8(13):2511–22. 10.7150/jca.18161 28900489 PMC5595081

[29] Wang L Fu D Qiu Y Xing X Xu F Han C Genome-wide screening and identification of long noncoding RNAs and their interaction with protein coding RNAs in bladder urothelial cell carcinoma. Cancer Lett (2014). 349(1):77–86. 10.1016/j.canlet.2014.03.033 24705305

[30] Mootha VK Lindgren CM Eriksson K-F Subramanian A Sihag S Lehar J PGC-1α-responsive genes involved in oxidative phosphorylation are coordinately downregulated in human diabetes. Nat Genet (2003). 34(3):267–73. 10.1038/ng1180 12808457

[31] Cantini L Isella C Petti C Picco G Chiola S Ficarra E MicroRNA-mRNA interactions underlying colorectal cancer molecular subtypes. Nat Commun (2015). 6:8878. 10.1038/ncomms9878 27305450 PMC4660217

[32] Menzl I Zhang T Berger-Becvar A Grausenburger R Heller G Prchal-Murphy M A kinase-independent role for CDK8 in BCR-ABL1(+) leukemia. Nat Commun (2019). 10(1):4741. 10.1038/s41467-019-12656-x 31628323 PMC6802219

[33] Lee K-S Kim S-W Lee H-S. Orostachys japonicus induce p53-dependent cell cycle arrest through the MAPK signaling pathway in OVCAR-3 human ovarian cancer cells. Food Sci Nutr (2018). 6(8):2395–401. 10.1002/fsn3.836 30510740 PMC6261214

[34] Liu T-Q Wang G-B Li Z-J Tong X-D Liu H-X. Silencing of Rac3 inhibits proliferation and induces apoptosis of human lung cancer cells. Asian Pac J Cancer Prev (2015). 16(7):3061–5. 10.7314/apjcp.2015.16.7.3061 25854406

[35] Wang G Wang H Zhang C Liu T Li Q Lin X Rac3 regulates cell proliferation through cell cycle pathway and predicts prognosis in lung adenocarcinoma. Tumor Biol (2016). 37(9):12597–607. 10.1007/s13277-016-5126-7 27402308

[36] Zhu Y Dai B Zhang H Shi G Shen Y Ye D. Long non-coding RNA LOC572558 inhibits bladder cancer cell proliferation and tumor growth by regulating the AKT-MDM2-p53 signaling axis. Cancer Lett (2016). 380(2):369–74. 10.1016/j.canlet.2016.04.030 27130667

[37] Zhang ZF Zhang HR Zhang QY Lai SY Feng YZ Zhou Y High expression of TMEM40 is associated with the malignant behavior and tumorigenesis in bladder cancer. J Transl Med (2018). 16(1):9. 10.1186/s12967-017-1377-3 29351801 PMC5775579

