# Supplementary material for: Rac3 Expression and its Clinicopathological Significance in Patients With Bladder Cancer
Source: Pathol Oncol Res. 2021 Mar 30;27:598460. doi: 10.3389/pore.2021.598460 (PMC8262164; doi:10.3389/pore.2021.598460)
Supplement: Supplementary file 1 [file Table1.DOCX]

Supplementary Table 1 Clinical characteristics of patients with BC in the Lee Bladder dataset

| Characteristics |  | Total | % | Rac3 expression values |
| --- | --- | --- | --- | --- |
| Age at diagnosis (y) |  | 56 (24~88) |  | 8.24 (7.02-11.04) |
| Gender | Male | 135 | 81.82 | 8.23 (7.02-10.65) |
|  | Female | 30 | 18.12 | 8.32 (7.14-11.04) |
| Grade | High | 60 | 36.36 | 8.44 (7.10-11.04) |
|  | Low | 105 | 63.64 | 8.22 (7.02-9.71) |
| T stage | Ta | 23 | 13.94 | 8.31 (7.54-10.13) |
|  | T1 | 80 | 48.48 | 8.28 (7.14-10.04) |
|  | T2 | 32 | 19.39 | 8.23 (7.10-9.97) |
|  | T3 | 19 | 11.52 | 8.15 (7.41-9.26) |
|  | T4 | 11 | 6.67 | 8.27 (7.02-9.03) |
| N stage | N0 | 149 | 90.30 | 8.23 (7.10-11.04) |
|  | N1 | 8 | 4.85 | 8.06 (7.02-8.99) |
|  | N2 | 6 | 3.63 | 8.90 (8.12-9.97) |
|  | N3 | 1 | 0.61 | 8.53 |
|  | Nx | 1 | 0.61 | 9.26 |
| M stage | M0 | 158 | 95.76 | 8.24 (7.10-11.04) |
|  | M1 | 7 | 4.24 | 8.53 (7.02-9.03) |

Abbreviations: BC, bladder cancer; T, tumor invasion; N, lymph node metastasis; M, metastasis.
