# Supplementary material for: Rac3 Expression and its Clinicopathological Significance in Patients With Bladder Cancer
Source: Pathol Oncol Res. 2021 Mar 30;27:598460. doi: 10.3389/pore.2021.598460 (PMC8262164; doi:10.3389/pore.2021.598460)
Supplement: Supplementary file 2 [file Table2.DOCX]

Supplementary Table 2 Clinical characteristics of patients with BC in the Sanchez-Carbayo Bladder dataset

| Characteristics |  | Total | % | Rac3 expression values |
| --- | --- | --- | --- | --- |
| Age at diagnosis (y) |  | 60 (34~86) |  | 27.2 (4.40-349.90) |
| Gender | Male | 74 | 70.48 | 22.10 (5.70-349.90) |
|  | Female | 31 | 29.52 | 35.80 (4.40-152.20) |
| Grade | 2 | 20 | 19.05 | 22.90 (5.30-152.20) |
|  | 3 | 82 | 78.10 | 23.80 (4.40-349.90) |
|  | Unknown | 3 | 2.85 | 13.6 (20.00-29.90) |
| Stage | Tis | 1 | 0.95 | 12.70 |
|  | Ta | 2 | 1.90 | 12.70 (5.30-21.00) |
|  | T1 | 25 | 23.81 | 21.00 (9.40-120.30) |
|  | T2 | 11 | 10.48 | 27.10 (14.10-99.50) |
|  | T3 | 52 | 49.52 | 21.90 (4.40-349.90) |
|  | T4 | 11 | 10.48 | 43.90 (16.20-119.00) |
|  | NT | 3 | 2.86 | 13.6 (20.00-29.90) |
| Positive lymph nodes | No | 76 | 72.38 | 22.70 (4.40-349.90) |
|  | Yes | 29 | 27.62 | 33.80 (10.30-156.30) |

Abbreviations: BC, bladder cancer; T, tumor invasion.
