# Supplementary material for: Rac3 Expression and its Clinicopathological Significance in Patients With Bladder Cancer
Source: Pathol Oncol Res. 2021 Mar 30;27:598460. doi: 10.3389/pore.2021.598460 (PMC8262164; doi:10.3389/pore.2021.598460)
Supplement: Supplementary file 4 [file Table4.DOCX]

Supplementary Table 4 Clinical characteristics of patients with BC in the GSE32894 dataset

| Characteristics |  | Total | % |
| --- | --- | --- | --- |
| Age at diagnosis (y) |  | 58 (20~96) |  |
| Gender | Male | 80 | 25.97 |
|  | Female | 228 | 74.03 |
| Grade (WHO 1999) | G1 | 48 | 15.58 |
|  | G2 | 103 | 33.44 |
|  | G3 | 154 | 50.01 |
|  | G4 | 1 | 0.32 |
|  | Gx | 2 | 0.65 |
| Stage (WHO 1999) | Ta | 116 | 37.66 |
|  | T1 | 97 | 31.49 |
|  | T2 | 85 | 27.60 |
|  | T3 | 7 | 2.27 |
|  | T4 | 1 | 0.32 |
|  | Tx | 2 | 0.66 |

Abbreviations: BC, bladder cancer; G, grade; T, tumor invasion.
